# Chitosan nanoparticles suspension can minimize enamel loss after *in vitro* erosive challenge

**DOI:** 10.1590/1678-7757-2024-0445

**Published:** 2025-04-18

**Authors:** Renata Cristina Sobreira FRANÇA, Rebeca Tibau Aguiar DIAS, Ranam Moreira REIS, Frederico Barbosa de SOUSA, Hugo Lemes CARLO, Rogerio Lacerda dos SANTOS, Fabíola Galbiatti de CARVALHO

**Affiliations:** 1 Universidade Federal da Paraíba Programa de Pós-Graduação em Odontologia João Pessoa Paraíba Brasil Universidade Federal da Paraíba (UFPB), Programa de Pós-Graduação em Odontologia, João Pessoa, Paraíba, Brasil.; 2 Universidade Estadual de Campinas Programa de Pós-Graduação em Odontologia Piracicaba São Paulo Brasil Universidade Estadual de Campinas (UNICAMP), Programa de Pós-Graduação em Odontologia, Piracicaba, São Paulo, Brasil.; 3 Universidade Federal de Uberlândia Departamento de Dentística e Materiais Dentários Uberlândia Minas Gerais Brasil Universidade Federal de Uberlândia (UFU), Departamento de Dentística e Materiais Dentários, Uberlândia, Minas Gerais, Brasil.; 4 Universidade Federal de Juiz de Fora Governador Valadares Minas Gerais Brasil Departamento de Odontologia, Universidade Federal de Juiz de Fora, Campus Governador Valadares (UFJF/GV), Governador Valadares, Minas Gerais, Brasil.; 5 Universidade Federal de Uberlândia Departamento de Odontopediatria Uberlândia Minas Gerais Brasil Universidade Federal de Uberlândia (UFU), Departamento de Odontopediatria, Uberlândia, Minas Gerais, Brasil.

**Keywords:** Chitosan, Nanoparticles, Erosion, Enamel

## Abstract

Chitosan nanoparticles suspension (ChNPs) showed antimicrobial effects in the oral cavity, but its effects on enamel erosion prevention remain little explored.

**Objective:**

This study synthesized ChNPs and evaluated their effect on enamel after erosive challenge.

**Design:**

ChNPs were synthesized by ionic gelation and characterized using scanning electron microscopy (SEM), infrared spectrophotometry (FTIR), dynamic light scattering methods (DLS) and zeta potential (ZP). In total, 56 human enamel blocks were divided into four groups (n=14/group): (i) ChNPs suspension (4.4mg/mL); (ii) 0.05% sodium fluoride solution (NaF); (iii) chitosan solution (5.0 mg/mL); and (iv) distilled water. After incubation in freshly collected human saliva (3h), the samples were exposed to erosive challenge in 1% citric acid (90s) and remineralizing solution (2h) performed four times a day. After the 1^st^ and 4^th^ acid exposures, solutions were applied for 2 min. After 7 days, % Vickers surface hardness change (% SMH) was obtained using 300 g load applied for 15s. Enamel surface loss was evaluated using optical profilometer by subtracting the final profile values from baseline ones. Data were analyzed by ANOVA and post-hoc T tests (α=0.05). Surface topography was obtained by optical profilometer.

**Results:**

SEM revealed the formation of spherical nanoparticles. DLS showed nanoparticles with 85.7±10.5 nm diameter and ZP value of +45.5±5.4mV. Enamel surface loss was significantly lower in ChNPs and NaF groups, exhibiting a less rough surface in the treated areas. ChNPs, NaF and chitosan groups showed lower % SMH values.

**Conclusions:**

ChNPs suspension minimized enamel loss after *in vitro* erosive challenge and appears to be a promising material for enamel erosion prevention.

**Figure:**
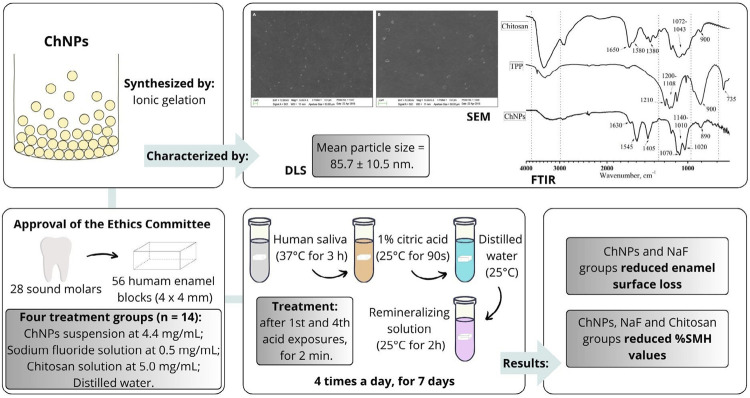
The article is a study produced from a master thesis available at: https://repositorio.ufpb.br/jspui/handle/123456789/11637

## Introduction

Dental erosion presents as pathological and progressive tooth structure loss due to chemical dissolution caused by intrinsic or extrinsic acids of non-bacterial origin.^1^ Teeth exposure to fluoride or other protective or remineralizing agents is one strategy among others to prevent erosion processes and treat early stages of erosive lesions.^1 , 2^

Fluoride agents can reduce tooth dissolution and increase dental resistance to erosive acids in some cases.^1^ These agents can contain a monovalent fluoride compound like sodium fluoride (NaF) or fluoride in combination with polyvalent metal ions (e.g., stannous and titanium tetrafluoride).^3 , 4 , 5^ Their protective effect is associated with the precipitation of loosely bound (non-specifically adsorbed) non-stoichiometric calcium fluoride-like (CaF_2_-like) material onto the eroded tooth surface,^6^ or incorporation of metal ions into the tooth upper surface and formation of a glaze-like layer that acts as an acid-resistant diffusion barrier.^3^

Dissolution of CaF_2_ deposits under highly acidic erosive conditions^7^ and the lacking substantivity of fluoridated agents in the oral cavity, especially when delivered via daily application vehicles,^8^constitutes major factors in driving research on biopolymers for preventing and treating early erosive lesions.^2^ Biopolymers can potentially enhance active principles retention within the oral cavity due to their capacity for film formation and affinity for dental structures by creating a protective barrier against erosive acids.^2 , 5^

Chitosan is a biopolymer and an *N* -deacetylated product derived from chitin, a natural compound found in the shells and exoskeleton of crustaceans.^9^ Besides applications in the pharmaceutical industry, it has shown potential benefits in dentistry by providing protection against enamel demineralization caused by caries^10^ and erosion.^11 , 12^ Usually, chitosan is incorporated into experimental toothpastes or solutions enriched with monovalent fluoride compounds or polyvalent metal ions capable of preventing dental erosion.^2 , 11^ However, studies show that chitosan solution alone can form a film or pellicle on tooth surface by adsorption and thus penetrate into the enamel and dentinal tubules of eroded structures.^13 , 14^

Interest in developing nanoscale systems from cationic polymers like chitosan nanoparticles has increased since biopolymers can favor molecular attractive forces formed by electrostatic interaction between positively charged chitosan and negatively charged surfaces. A cation originating from the amine group (NH_2_) in chitosan becomes protonated at low pH (NH_3_^+^) and interacts with negatively charged components, including proteins, anionic polysaccharides, and the negatively charged demineralized enamel surface covered by an acquired salivary pellicle.^10 , 13^ Following chitosan dissolution, the acidic pH can create gaps between the surface layer crystals; consequently, chitosan can engage with the enamel surface, forming a physical protection barrier against acid attacks.^10 , 13^

Chitosan nanoparticles (ChNPs) can be synthesized through several methods. Ionic gelation using polyanions such as sodium tripolyphosphate (TPP) stands out for its easy and quick execution. ChNPs formation via ionic gelation, which involves combining cationic chitosan and TPP anionic compounds, produces hydrophobic precipitates in the form of gel-like nanoparticles.^10^These are stabilized by means of three-dimensional cross-linked Lewis acid-base interactions between anionic (P-O⁻) and cationic (NH₃⁺) groups, facilitating their precipitation from an aqueous solution.^10^ This nanoparticle suspension serves as a controlled drug delivery system, relying on ionic dissociation during dilution, besides exhibiting mucoadhesive properties and forming a physical barrier that enables the gradual and controlled release of active compounds into the oral epithelium (including the alveolar mucosa, cheek, and gingiva) and the enamel surface.^10 , 13 , 15^

Interestingly, the antimicrobial effects of chitosan-based nanoparticles and microparticles in the oral cavity have been previously investigated,^15 , 16^and chitosan has been used in commercial formulations (toothpastes and solutions) to prevent or refrain erosive wear.^12 , 13^ However, ChNPs effects on enamel erosion prevention remain unexplored.

Thus, this study synthesized chitosan nanoparticles suspension and characterized it using scanning electron microscopy (SEM), Fourier transformation infrared spectrophotometry (FTIR), dynamic light scattering (DLS) and zeta potential (ZP). The in vitro effect of ChNPs suspension on preventing human enamel erosion was assessed by measuring enamel loss using an optical profilometer and evaluating enamel softening through microhardness testing. Our null hypothesis posited that ChNPs suspension would be unable to minimize tissue loss and change in hardness of the eroded enamel.

## Methodology

### ChNPs suspension synthesis and characterization

A total of 5 mg/mL low molecular weight chitosan (107kDa, 75-85% degree of deacetylation) (Sigma-Aldrich, St. Louis, USA) was dissolved in a solution containing 1% acetic acid (w/v) (Química Moderna, Barueri, São Paulo, Brazil) and kept under stirring conditions at room temperature for 24 h, totaling a final chitosan solution volume of 10 mL.^10^ Subsequently, the solution was filtered through membrane filters with 5.0 μm and 0.8 μm pore sizes. ChNPs suspension was synthesized using ionic gelation with added sodium tripolyphosphate (TPP).^10^ A total of 2.4 mg/mL aqueous solution of TPP (Sigma-Aldrich, St. Louis, USA) was prepared separately, of which 3 mL was added to the 10 mL chitosan solution under agitation at 6000 rpm and room temperature using a continuous infusion pump at a 60 mL/h rate.^10^ Following the chemical formula of mixing solutions, the final ChNPs suspension concentration was 4.4 mg/mL.^10^ pH of the suspension was adjusted to 5.5 by adding NaOH to match the pH value of certain commercial mouthwashes^17^ and monitored for 7 days.

ChNPs were examined by scanning electron microscopy (SEM), Fourier transformation infrared spectrophotometry (FTIR), dynamic light scattering (DLS) and zeta potential (ZP).

For SEM analysis, a 50 µL aliquot of the ChNPs suspension was dripped onto carbon tape mounted on aluminum stubs and stored in a desiccator for 24 h. Stubs were sputtered-coated with gold under vacuum (Balzers-SCD 050 Sputter Coater, Balzers, Liechtenstein). SEM was performed using a LEO 1430 scanning electron microscope (Zeiss Inc, Thornwood, NY, USA). Nanoparticle formation and morphology were observed at 500 X and 1000 X magnification.

FTIR (IRPrestige-21, Shimadzu, Kyoto, Japan) is a spectroscopic technique used to obtain infrared spectra of substances for identification of chemical bonds and functional groups.^15^ Solid samples of chitosan and TPP were diluted in KBr:sample at a ratio of 100:1 mg and then pressed to form a pellet of approximately 1 cm in diameter, which were subsequently subjected to analysis by infrared beam transmittance. A droplet of ChNPs suspension was put on the crystal in attenuated total reflection (ATR) mode for analysis. FTIR spectra were obtained at a frequency range of 400 – 4000 cm^-1^, with a 4 cm^-1^ resolution and average of 20 scans. Results were analyzed on Origin Pro 8 (Northampton, MA, USA).

Dynamic light scattering (DLS) is an established and precise measurement technique for characterizing particle sizes in suspensions. Zeta potential (ZP) represents the electrical potential at the slipping plane of particles suspended in a fluid according to their surface charge.^18^Particles present in a liquid typically form an electrical double layer due to the interaction between particle surface charge and the existing ions. This surface charge attracts oppositely charged ions, creating a structured layer around the particle. As it moves through the liquid so does, partially, the electrical double layer up to a boundary known as slipping plane. The electrical potential measured at this point is the zeta potential. ZP reflects the degree of electrostatic repulsion or attraction between particles and is a key factor for determining the stability of colloidal systems.^18^

Hydrodynamic diameter ( *Dh* ) and zeta potential (ZP) of the nanoparticles were measured using a Zeta Sizer Nano ZS light scattering photometer (Malvern Panalytical, Malvern, UK). Immediately after synthesis, ChNPs suspension was transferred into a specific square polyethylene cuvette for particle size measurement under monochromatic light (10 mW He-Ne laser, 632.4 nm wavelength). Scattered light intensity was measured at a 90° angle. *Dh* values were measured in quintuplet independently, mean of 30 counts, at 25°C after 30 s of equilibration time.^10^ZP measurements were obtained via electrophoretic mobility determined by laser Doppler microelectrophoresis at a scattering angle of 173° using a zeta potential meter (Zeta Sizer Nano ZS, Malvern, UK). ChNPs suspension aliquots were placed onto the disposable folded capillary cell of the device (DPS1060) and ZP values were measured 5 times independently, mean of 30 counts, at 25°C after 60 s of equilibration time.^9^

### Sample preparation and study groups

Sample size for testing the eroded enamel microhardness treated with agents containing chitosan (n = 20) was calculated using ANOVA based on the following parameters: 80% power, 5% significance level, effect size “f” of 0.68, and degrees of freedom (number of population means -1) of 2 (Table 8.4.4 in Cohen).^19^ For mineral loss testing, the sample size (n = 14) was calculated using ANOVA based on a 5% significance level, effect size “f” of 1.3, and power of 80%.

A total of 28 healthy molars were obtained after approval of the Federal University of Paraíba Research Ethics Committee (Protocol No. 46087215.2.0000.5188), stored in 0.1% thymol at 4°C and used within 1 month after extraction. A 4 mm x 4 mm window was drawn on the proximal surfaces of each tooth, totaling an enamel area of 16 mm^2^, and two enamel blocks (one from each proximal surface) were obtained using a double-sided disc mounted on a micromotor handpiece under cooling. Each block was fixed in acrylic resin (Vipi Flash, Pirassununga, SP, Brasil) and the enamel surface was serially polished for 1 min under constant irrigation for each silicon carbide abrasive paper used (400, 600, and 1200 grits) and for alumina suspension (1 μm) (Erios Corp, São Paulo, SP, Brazil). Samples were cleaned with distilled water for 10 min in ultrasonic bath (Ultrasonic Cleaner, model USC1400, Unique Ind. and Com. Ltda, São Paulo). Presence of surface cracks and imperfections was examined using stereomicroscope (magnification of x40; Nikon 88286, Tokyo, Japan); if found, the sample was replaced. The acrylic resin around the enamel block of each sample was coated with two layers of nail varnish (Colorama, CEIL Ltda, SP, Brazil).

Samples were arbitrarily divided into four groups (n=14) according to the agent applied as follows:

Group 1 – ChNPs suspension at 4.4 mg/mL – pH 5.5;

Group 2 – Sodium fluoride solution at 0.5 mg/mL (0.05%) – pH 6.0 (positive control);

Group 3 – Chitosan solution at 5.0 mg/mL – pH 5.5;

Group 4 – Distilled water (negative control) – pH 6.0.

### Profilometer analysis

For tissue loss measurement, the baseline profiles of all samples were obtained using an optical profilometer (Taylor Hobson - CCI MP, West Chicago, Illinois, USA). Two parallel grooves were made in the resin, close to the sample, to produce orientation guides during profile readings.^20^ Three measurements were taken at 0.2 mm intervals at the center of each specimen. Each scan length covered the reference area of acrylic resin and enamel measuring 0.8 mm long (X) x 0.8 mm wide (Y).

### Agent application and erosive challenge

After incubating the samples in freshly collected human saliva (20 mL/8 enamel samples) at 37°C for 3 h under constant shaking,^21^ the stimulated saliva was collected in an ice cooled tube from one healthy adult donor (stimulated salivary flow rate 2.35 mL/min) by chewing on a piece of paraffin pellet (Fluka, Sigma-Aldrich, Munich, Germany) for 30 min at least 1 h after having consumed any food or drink.^21^ Approval was obtained from the local Ethical Committee (CAAE: 46087215.2.0000.5188).

**Figure 1:**
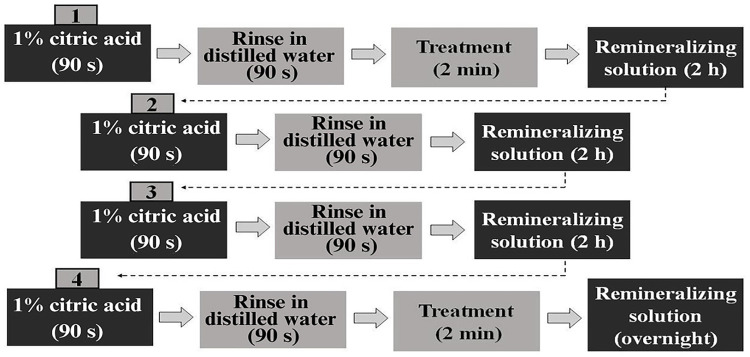
Flowchart of the agent application during erosive challenge

Subsequently, the samples underwent erosive challenge with 1% citric acid monohydrate solution (Sigma-Aldrich, St. Louis, USA) (pH 3.6) according to Souza et al.^22^ (2014) and using remineralizing solution performed by a single blinded operator (RCSF). Samples were immersed in 1% citric acid (30 mL per sample) at 25^o^ C (room temperature) for 90 s, rinsed in distilled water for 90 s, and then immersed for 2 h in remineralizing solution four times a day for 7 days. After the 1st and 4th acid exposures, treatment was performed using a pipette to apply the respective agents on enamel surface for 2 min (1 mL/sample),^23^ and any excess was removed using cotton swabs. Samples were re-immersed in the remineralizing solution and stored overnight. Figure 1 shows the daily cycling sequence.

Remineralizing solution consisted of calcium (Ca^2^) – 1.5 mmol L^-1^, phosphate – 0.9 mmol L^-1^, and potassium chloride (KCl) 150 mmol L^-1^ in 0.1 mol L^-1^ of TRIS buffer, pH 7.0.^24^

### Surface microhardness (SMH) measurements

Hardness measurements were performed pre- and post-erosive challenge using HMV-2 microdurometer (Shimadzu, Kyoto, Japan), with Vickers indenter (300 g load applied for 15 s). In total, 5 indentations were made at 100 μm intervals from each other. Measurements were taken from both the control (initial) and experimental sides (after erosive challenge). Percentage of change in surface hardness ( **%** SMH) was calculated using the following formula:^25^


$$\%SMH=100\times \left[\left(SMH_{\text{post-challenge }}-SMH_{\text{initial }}\right)/SMH_{\text{initial }}\right]$$

### Tissue loss measurement and Surface topography

After the erosive challenge, a scalpel blade was used to remove the nail varnish from the acrylic resin portion, and the surface of each sample was scanned using an optical profilometer (Taylor Hobson - CCI MP, West Chicago, Illinois, USA). Samples were positioned in a custom-made setting device to ensure sample placement after agent application and erosive challenge. Final profiles were obtained using the same parameters as those employed for baseline profiles. Tissue loss (μm) was calculated by subtracting the final profile values from baseline ones using the previously described grooves as guides. Values were treated with Taly Map Lite 7.2 (64-bit version).

Surface topography analyses were performed using a 0.25 mm cut-off and a 20x magnification lens, operating at a constant speed of 1 mm/s in XYZ mode and resolution of 512 × 512 pixels. A Gaussian filter (FALG), as specified in ISO 16610-61, was applied. Each scan covered the central slab area, measuring 0.8 mm in length (X-axis) and 0.8 mm in width (Y-axis). Data were processed using Taly Map Lite 7.2 (64-bit version).

### Statistical analysis

FTIR, SEM, DLS and ZP data were descriptively analyzed. For both experiments (profilometric and microhardness analyses), we tested the null hypothesis that treatment (in 4 levels) would not affect the outcome. All groups were tested relative to data normality by calculating kurtosis and skewness. Those with a variation of ± 2 from the optimal value (-2 to 2 in skewness; 1 to 5 in kurtosis) were considered normally distributed.^26^ Homogeneity test of variances for both ANOVA and post-hoc tests was performed by calculating the variance ratio, defined as the ratio of the largest variance to the smallest variance of groups. Variances were considered homogeneous when the ratio was ≤ 3.^27^ Comparisons among groups were estimated by one-way ANOVA, whereas comparisons between pairs of groups were calculated by and post-hoc T tests. Significance level was set at 5% (2-tailed for T tests). Effect size, its 95% confidence interval and power were also calculated.^19^ Bonferroni correction for type I error was not performed as all tests were planned in advance during study designing.^28^

## Results

### ChNPs suspension characterization

**Figure 2:**
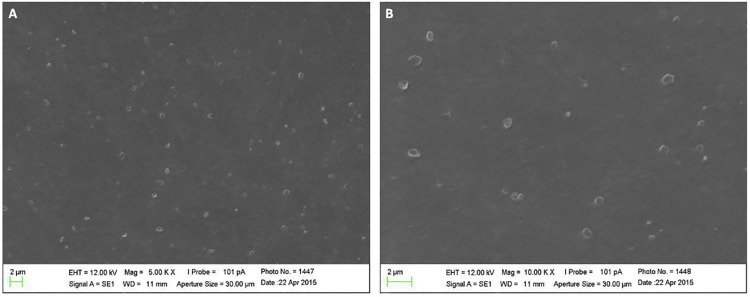
Scanning electron microscopy photomicrograph images showing chitosan nanoparticle formation at 500 X (A) and 1000 X (B) magnification. Reference bar = 2 μm

SEM images showed the formation of dispersed spherical nanoparticles ( Figures 2 A and B ).

**Figure 3:**
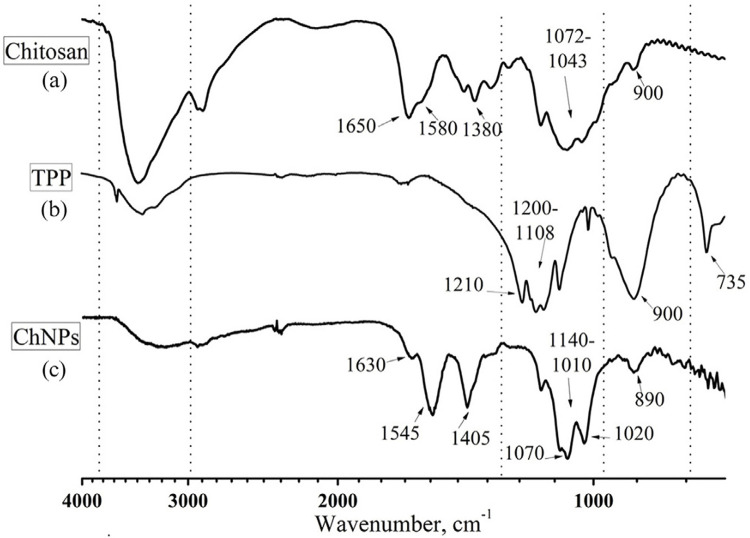
Infrared spectroscopy images (FTIR). (a) Chitosan powder; (b) TPP powder; (c) Chitosan nanoparticles (lyophilized).

Figure 3 presents the FTIR absorption values for chitosan, TPP and ChNPs.

**Figure 4:**
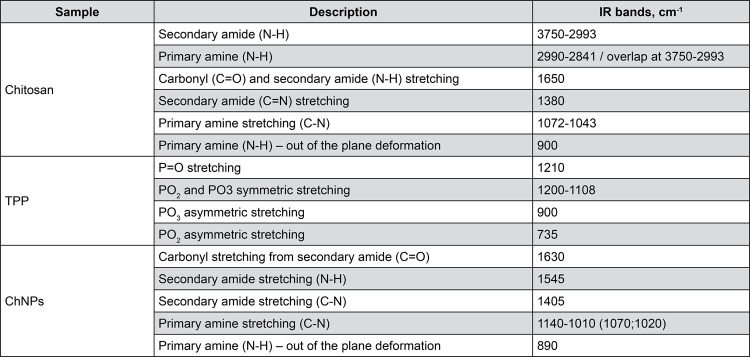
Description of Fourier-transform infrared spectroscopy (FTIR) absorption of chitosan, TPP and ChNPs.

Figure 3 and Figure 4 describe the FTIR bands. Chitosan showed an overlapping region at 3750 – 2993 cm^-1^related to N-H vibration of the secondary amide and primary amine. Primary amine stretching (N-H) associated with the presence of two hydrogens was found at 2990 – 2841 cm^-1^. Secondary amide carbonyl stretching appeared at 1650 – 1580 cm^-1^. Absorption values of 1380 cm^-1^, 1072 – 1043 cm^-1^ and 900 cm^-1^ were related to secondary amide C-N stretching, primary amine C-N stretching and primary amine N-H out of the plane deformation, respectively. TPP infrared spectrum found PO_2_ and PO_3_ symmetric stretching band at 1220-1108 cm^-1^, P=O stretching at 1210 cm^-1^, and PO_2_ and PO_3_ asymmetric stretching at 900 cm^-1^ and 735 cm^-1^, respectively. ChNPs absorption values are be observed at 1630 cm^-1^, 1545 cm^1^ and 1405 cm^-1^ related to C=O, N-H, and C-N stretching of the secondary amide. At 1140-1010 cm^-1^ we note a primary amine region due to C-N stretching, with peaks related to two N-H bonds in different environments. C-H out of the plane deformation occurred at 890 cm^-1^.

DLS obtained a mean particle size of 85.7 ± 10.5 nm. ZP value was +45.5 ± 5.4mV. pH of the ChNPs suspension remained stable for 7 days (5.5 ± 0.3).

### Tissue loss measurement and Surface topography

Our null hypotheses that surface treatment would not affect tissue loss measurement and % SMH were rejected (ANOVA): (i) tissue loss measurement, P< 0.00001, effect size η2 (eta squared) of 62.86% (percentage of variances explained by treatment), and power > 99.99%; and (ii) % SMH, P< 0.00001, effect size η2 (eta squared) of 31.46 % (percentage of variances explained by treatment), and power of 99.95%. Tables 1 and 2 summarize the comparisons between pairs of groups.

Post-hoc paired T test revealed an enamel surface loss significantly lower in the ChNPs and NaF groups after erosive challenge, without significant difference between them. Chitosan solution showed higher values of enamel surface loss with no difference in comparison with control ( Table 1 ).

**Table 1:** Results of changes in tissue loss measurement (μm) of enamel after erosive challenge, described as mean (standard deviation - SD).

	Groups			
	ChNPs	NaF	Chitosan	Water
Mean (SD)	5.53 (2.39)^A^*	5.19(1.69)^A^	9.49 (2.48)^B^	10.91 (1.00)^B^
*Post hoc paired T tests*				
Groups compared	**P value**	**Hedge’s g**	**95% CI**	**Power**
ChNPs X NaF	0.6835	0.164	0.975/ -0.647	0.056
ChNPs X Chitosan	0.0004	1.628	2.563/ 0.694	0.976
ChNPs X Water	1.32 x 10-7	2.941	4.109/ 1.773	0.999
NaF X Chitosan	3.61 x 10-5	2.028	3.025/ 1.032	0.998
NaF X Water	5.11 x 10-10	4.119	5.549/ 2.689	0.999
Chitosan X Water	0.082	0.753	1.591/-0.085	0.443

* Same capital letters indicate no statistically significant difference among the Groups (p> 0.05 by ANOVA, followed by post-hoc T tests).

**Figure 5:**
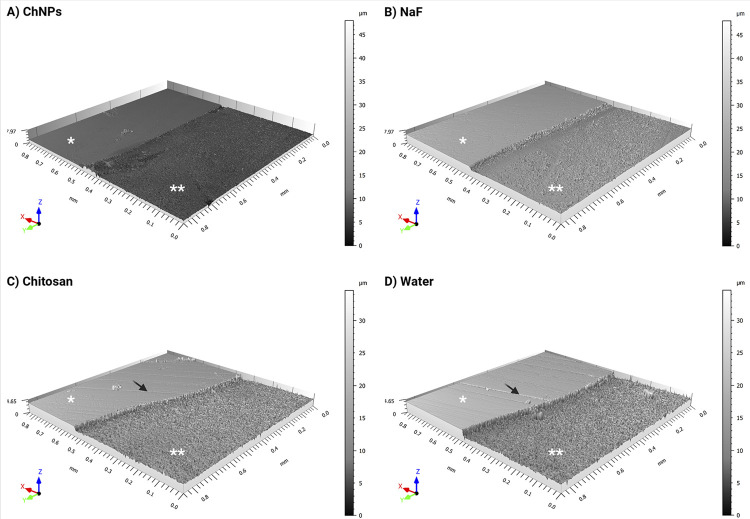
Enamel surface topography by optical profilometer of healthy and treated areas: (A) ChNPs suspension; (B) 0.05% NaF; (C) Chitosan solution and (D) Distilled water. * indicates the healthy area and ** denotes the treated area (after agent application and erosive challenge). Healthy areas show some scratches from sample polishing (→).

Figure 5 illustrates the enamel surface topography of healthy and treated sample areas in each group. All treated exhibited a rough surface, however the chitosan and water groups showed a rougher surface (Figures 5C and 5D) than the ChNPs and NaF groups (Figures 5A and 5C). Additionally, the healthy areas of the chitosan and water groups presented some scratches caused by specimen polishing (Figures 5C and 5D).

### Microhardness analysis

For surface microhardness, all agents showed significant decrease in SMH values after erosive challenge ( *P* = 0.001). % SMH presented no significant difference between the ChNPs, NaF and chitosan groups, but they all had lower % SMH when compared with control (water) ( Table 2 ).

**Table 2:** Results of initial and final (post-erosive challenge) surface microhardness measurements (SMH) and the percentage of change in surface hardness (% SMH) of enamel after erosion, described as mean (standard deviation - SD).

	Groups							
	ChNPs		NaF		Chitosan		Water	
	Initial	Final	Initial	Final	Initial	Final	Initial	Final
Mean (SD) SMH values	369.7	303.6	369.5	294.6	370.0	318.0	369.6	258.3
initial and final	(21.1)^A^*	(28.7)^B^	(21.4)^A^	(37.4)^B^	(18.0)^A^	(51.4)^B^	(25.2)^A^	(41.9)^B^
Mean (SD) % SMH	20.23 (7.44)^b^**		21.44 (9.45)^b^		22.86 (8.28)^b^		34.54 (10.50)^a^	
*Post hoc paired T tests for %SMH*								
Groups compared	**P value**		**Hedge’s g**		**95% CI**		**Power**	
ChNPs X NaF	0.6845		0.145		0.841/ -0.551		0.058	
ChNPs X Chitosan	0.2981		0.334		0.978/ -0.311		0.169	
ChNPs X Water	1.45 x 10-5		1.572		2.305/ 0.840		0.997	
NaF X Chitosan	0.6471		0.161		0.859/ -0.535		0.064	
NaF X Water	0.0005		1.300		2.064/ 0.537		0.957	
Chitosan X Water	0.0004		1.235		1.934/0.537		0.966	

*Same capital letters indicate no significant difference between initial and final SMH values for each agent by ANOVA and post hoc paired T tests (p< 0.05).

**Same lower-case letters indicate no significant difference among groups for %SMH by ANOVA and post hoc paired T tests (p< 0.05).

## Discussion

Our null hypothesis was rejected since ChNPs suspension minimized tissue loss and changes in surface hardness after erosive challenge.

ChNPs formation via ionic gelation was successfully performed and confirmed by SEM images, FTIR and DLS analysis. SEM images revealed spherical nanoparticle formation (Figures 2 A and B). FTIR analysis showed the intermolecular interactions between chitosan and TPP, confirming the crosslink of chitosan ammonium groups with TPP molecules by two new bands at 1632 and 1547 cm^-1^formed for ChNPs group.^29 , 30^

DLS also observed interaction between chitosan and TPP molecules. ChNPs had a mean particle size of 85.7 ± 10.5 nm which is consistent with findings from other studies that employed ionic gelation with TPP for ChNPs synthesis.^10 , 15^ Several factors can influence the size of chitosan particles, including synthesis method, pH of the media, and the concentration and molecular weight of chitosan.^31^ Sreekumar, et al.^32^ (2018) found that for a given chitosan-to-TPP molar ratio, the average hydrodynamic diameter of the particles formed is highly dependent on the initial chitosan concentration. Additionally, the degree of chitosan acetylation was the second most significant factor influencing the particle-forming capability of the system. These parameters explain most of the inter-study differences concerning nanoparticle size. The positive ZP values found (+45.5 ± 5.4mV) indicate the presence of free amino groups in chitosan as titration with TPP anions has previously been shown to reduce ZP by neutralizing cationic groups.^15^ In our study the pH of ChNPs remained unaltered for 7 days, suggesting a stable ChNPs suspension. The pH of the media can affect relevant physicochemical characteristics of ChNPs suspensions and their stability by changing parameters like particle hydrophobicity, wettability, and surface homogeneity.^33^

We used 1% citric acid (pH 3.6) for the erosive challenge to mimic orange juice,^11^ simulating the daily application of agents by patients (in the morning and evening) as in Souza, et al.^22^ (2014). Human saliva was applied to mimic the presence of acquired salivary pellicle on the enamel surface which plays an important role in preventing erosive demineralization.^34 , 35^ We collected stimulated saliva to reflect physiologically relevant conditions for erosion and remineralization processes that occur during eating, as it predominates in the oral cavity during mastication or food consumption. Moreover, stimulated saliva contains essential salivary proteins such as statherin and histatins, which play a crucial role in initiating acquired pellicle formation and protecting enamel.^35^Samples were pre-incubated for 3 h in human saliva, as outlined in Lussi and Carvalho^21^study (2015), because despite no significant difference observed in the protective ability of pellicles when comparing 24h and 7-day-old pellicles, its acid resistance appears to depend on its formation time.^34 , 35^ Specifically, a 2h pellicle dissolves from the enamel surface more rapidly than 6, 12, and 24h pellicles.^34^

Acquired salivary pellicle is known to be a bacteria-free film that covers dental structures and contains several proteins, including mucins, glycoproteins, and proline-rich proteins.^34^At the onset of salivary pellicle formation, some precursor proteins with strong affinity for hydroxyapatite develop electrostatic interactions with the enamel, forming the first layer of proteins (basal layer) that seems to provide the most significant protection against dental demineralization due to its highly electron-dense layer. The subsequent protein layers exhibit a much looser structure in comparison, consisting of globular proteins that bind to the precursor ones.^35^ The acid must therefore diffuse through the acquired pellicle before coming into contact with the enamel surface, enabling the pellicle to act as a diffusion barrier or semipermeable membrane that reduces and retards immediate interaction between acids and the enamel surface, protecting the tooth against demineralization.^34^However, studies have suggested that only mature pellicles, several days old, can prevent enamel demineralization.^34^

As we applied human saliva only prior to the erosive challenge to allow salivary pellicle formation, a single immersion in human saliva before the 7-day erosive challenge was insufficient to form a mature pellicle and to simulate the interaction between chitosan and salivary pellicle proteins. However, in clinical situations chitosan has a strong positive zeta-potential (+45.5 ± 5.4 mV) due to the amino group which allows a readily adsorption to surfaces with a strong negative zeta potential, like tooth enamel, via electrostatic forces.^9^ Mucins in the salivary pellicle can also adsorb onto negatively charged surfaces and, in the presence of chitosan, will adsorb onto the enamel to form firmly attached multilayers.^34^ This layer-by-layer build-up on enamel can be significantly acid resistant and provide better protection against erosive attacks.^2 , 5 , 11^

Results of tissue loss after erosive challenge showed different degrees of protection against enamel demineralization between the chitosan agents tested. ChNPs had significantly lower enamel loss compared with chitosan, which showed no enamel protection against the acid citric challenge ( Table 1 ). Figure 5 indicates that ChNPs and NaF groups exhibited a less rough surface in the treated areas (Figures 5A and 5C) compared with the chitosan and water groups (Figures 5C and 5D).

ChNPs’ mechanism of action on eroded enamel is unclear. Our findings suggest that in addition to chitosan adsorption onto the enamel structure,^36^ the diminute size of chitosan nanoparticles may have facilitated their interaction with the enamel surface, providing a greater protective effect on the enamel than the chitosan solution. Liu, et al.^37^ (2007) evaluated the adhesion of chitosan nanoparticles in a toothpaste and found that ChNPs have a better absorption and adhesion capacity on hydroxyapatite than the chitosan solution. We can also posit that chitosan nanoparticles are small enough to penetrate through the surface layer into the remaining spaces of demineralized prisms and bond onto the walls in a coordinated bond reaction via the chemical reaction between hydroxyapatite (metal ions such as Ca^2^) and chitosan (amino groups).^38^

NaF and ChNPs showed the lowest enamel loss values ( Table 2 ) with two daily applications and 0.05% NaF concentration. The different pH values of the solutions used to simulate the erosive challenge’s pH-cycle replicated the conditions experienced by patients frequently exposed to erosive attacks, with four 90-second erosion episodes per day. To counteract the erosive challenge, the solutions were applied twice daily. Results indicated that ChNPs suspension and 0.05% NaF solution showed potential to partially reduce enamel erosion. Although this finding is preliminary and based on an *in vitro* experiment, it suggests that patients suffering from erosion might benefit from daily application of ChNPs suspension and 0.05% NaF solution. Further studies are needed to validate this outcome.

Some studies have investigated the effectiveness of 0.05% NaF against enamel erosion,^2 , 7 , 22 , 39^ reporting that dissolution of the calcium fluoride (CaF_2_) deposits under high acidic erosive conditions could limit its effectiveness.^2 , 7 , 22^ Studies showed that NaF had an anti-erosive effect and was able to reduce surface loss after citric acid erosive challenge.^2 , 6 , 39^ However, depending on the magnitude of the acid challenge, a cumulative effect of several NaF applications could be needed to show some protection against erosion.^2^ NaF association with bioadhesive polymers in mouthwashes or the use of NaF mouthwash prior to brushing were tested and recommended to create a cumulative fluoride protective effect, leading to a significant reduction in enamel erosive wear.^2 , 7^

O’Toole, et al.^39^ (2015) found that 0.05% NaF mouthwash reduced enamel loss when applied after immersion in citric acid in the presence of saliva, corroborating our results. Saliva is capable of enhancing the action of sodium fluoride on enamel and minimizing erosive wear,^39^ as reported by *in situ* studies.^40 , 41^ In their discussion, O’Toole, et al.^39^ (2015) pointed out that the degree of conflicting data about NaF anti-erosive effect in laboratory investigations stems from the ideal timing for fluoride application; hence, literature findings indicate that when NaF was applied before the erosive challenge little or no anti-erosive effect was observed, whereas its application after erosion created a protective effect. *In vitro* results should not be extrapolated to *in vivo* situations, but the application timing of active agents in relation to the acid challenge (before or after) should be further investigated ( *in situ* or *in vivo* ) to evaluate the anti-erosive effect of NaF and biopolymer-containing mouthwashes.

After erosive challenge, all agents showed reduction in SMH values with no significant difference in % SMH between the ChNPs, NaF and chitosan groups ( Table 1 ), indicating that despite the reduction in enamel loss by profilometric analysis for ChNPs and NaF, the remaining structure was softer. O’Toole, et al.^39^ reported the same results (2015). This softened structure could contain within an intact enamel matrix which might improve the remineralization potential.^39^ Conversely, studies show that softened enamel is more susceptible to degradation from tooth brushing abrasion^11 , 12 , 42^ and this softened structure could result in further enamel wear. Another explanation for the lack of difference in % SMH between the experimental groups is that the indentations may have trespassed the softened (eroded) enamel layer into the underlying healthy enamel,^11^ hindering a difference in SMH between the agents tested.

Surface microhardness results showed that the citric acid challenge caused ‘softening’ of the enamel surface, and all agents investigated were able to prevent further enamel demineralization. Consequently, the likely mechanism of action of NaF, ChNPs and chitosan agents was related to a partial inhibition of the near-surface demineralization/enamel softening rather than enamel remineralization *per se.*

This study shows promising results for oral biology as the ChNPs suspension proved effective in minimizing enamel demineralization after an erosive challenge, emerging as a promising material for an oral formulation to be used for enamel erosion prevention. As this is a preliminary study, we point out some limitations. The *in vitro* acquired pellicle formed here cannot be directly extrapolated to *in vivo* acquired pellicle formation. Additionally, the human saliva used was collected from a single donor, and the pellicle was formed only prior to the erosive challenge. This approach was insufficient to enable the formation of a mature pellicle or to adequately simulate the interaction between chitosan and salivary pellicle proteins. Toothbrushing abrasion was not simulated but is an important factor to be considered in future studies due to the influence of mechanical forces on the durability of film-forming polymers on enamel surfaces.^7 , 23^ Moreover, our results can provide scope for further investigations using more clinically relevant experimental models ( *in situ* and *in vivo* studies). Evaluation of fluoride ions (NaF, amine fluoride or polyvalent metal ions) incorporation into ChNPs, forming a ChNPs/Fluoride ion complex, should also be investigated to analyze whether this combination improves enamel protection against dental erosion.

## Conclusions

Our findings provide a successful chitosan nanoparticles synthesis using ionic gelation. Chitosan nanoparticle suspension showed a protective effect on the tooth surface by reducing enamel loss and demineralization following *in vitro* erosive challenge. Chitosan and salivary pellicle proteins interaction requires further investigation during erosive challenges using mature salivary acquired pellicles in *in vitro, in situ* , or *in vivo* studies.

Data availability:

 The datasets generated during and/or analyzed during the current study are available from the corresponding author on reasonable request.
